# Dual-Camera Port Setup Strategies for Totally Endoscopic Aortic and Mitral Valve Surgery

**DOI:** 10.7759/cureus.86081

**Published:** 2025-06-15

**Authors:** Yoshihiro Goto, Atsuki Imagawa, Sho Takagi, Junji Yanagisawa, Yasuhide Okawa

**Affiliations:** 1 Cardiac Surgery, Toyohashi Heart Center, Toyohashi, JPN

**Keywords:** aorta, atrial fibrillation, cardiac surgery, endoscopic double-valve surgery, fibroelastoma, mitral valve, stenosis

## Abstract

Endoscopic cardiac surgery offers favorable early outcomes because of its minimally invasive nature. However, its application in combined aortic and mitral valve procedures remains limited owing to differences in optimal visualization angles and the technical complexity of the procedure. A septuagenarian woman (body surface area 1.2 m^2^) with severe mitral stenosis, atrial fibrillation, and aortic valve papillary fibroelastoma, who was wheelchair-bound, underwent endoscopic double-valve surgery. Preoperative computed tomography revealed that a single camera port did not provide optimal visualization. Therefore, we employed a dual-camera port setup to facilitate safe mitral valve replacement and aortic tumor resection. The patient recovered uneventfully and was discharged on postoperative day seven. The dual-camera port approach improves visualization and maneuverability in totally endoscopic double-valve surgery, offering a minimally invasive and effective alternative for complex cases, particularly in high-risk patients.

## Introduction

Endoscopic cardiac surgery has demonstrated favorable early outcomes owing to its minimally invasive nature [1-3]. Recent advancements in surgical technology, particularly the development of three-dimensional endoscopic systems and left atrial appendage closure devices, have substantially enhanced the safety, efficiency, and feasibility of totally endoscopic cardiac surgery. A recent study has demonstrated that 3D endoscopic systems reduce cardiopulmonary bypass time compared to that of conventional 2D systems due to their superior spatial resolution [4]. Similarly, the introduction of dedicated left atrial appendage occlusion devices has enabled safe and effective closure of the left atrial appendage [5]. These innovations have also contributed to shorter operative times. Frailty is associated with poorer outcomes following conventional cardiac surgery, and less invasive treatment modalities are therefore more appropriate for this population [6]. For patients who are not candidates for catheter-based interventions, surgical treatment remains necessary. Consequently, the indications for minimally invasive procedures have expanded to include even frail patient populations [7].

Recently, its application in mitral valve surgery has yielded excellent results [2]. However, totally endoscopic aortic valve surgery and combined procedures involving both the aortic and mitral valves, although reported with favorable outcomes in select institutions, have not yet been widely adopted [3]. A major reason for this is the difference in the optimal endoscopic approach angles required for each valve. In this report, we present a case of simultaneous totally endoscopic surgery for both the aortic and mitral valves in a frail, high-risk patient with limited physical capacity. The procedure was enabled by strategic modifications to the surgical setup, the use of advanced surgical tools such as a high-definition 3D endoscope and a left atrial appendage occlusion device enhanced intraoperative precision and safety. These integrated technological solutions contributed not only to improved maneuverability and visualization but also to the overall reduction in operative time. This case highlights how treatment strategies can be tailored to the patient's specific anatomical and clinical characteristics through the combination of individualized planning, minimally invasive techniques, and cutting-edge surgical technologies.

## Case presentation

The patient was a 77-year-old woman with a history of stroke and wheelchair use. In the present case, the patient presented with exertional dyspnea and fatigue. Physical examination revealed a diastolic murmur at the apex, and the patient was classified as New York Heart Association (NYHA) functional class III. Laboratory testing indicated an elevated B-type natriuretic peptide (BNP) level of 895 pg/mL that was consistent with decompensated heart failure.

**Figure 1:**
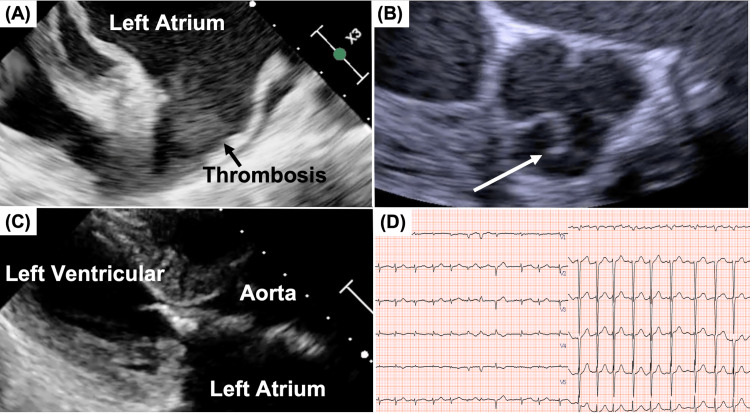
Preoperative Echocardiographic and Electrocardiographic Findings (A) Transesophageal echocardiographic view of the left atrial appendage. (B) Aortic valve in short-axis view. (C) Severe mitral stenosis with leaflet calcification. (D) Electrocardiogram showing atrial fibrillation. The white arrow indicates the papillary fibroelastoma

Cardiac evaluation for heart failure revealed severe mitral stenosis, atrial fibrillation, and papillary fibroelastoma of the aortic valve (Figure 1).

A multidisciplinary Heart Team consultation determined that catheter-based treatment was not suitable due to the presence of thrombi and other contraindications. Additionally, the patient’s long-standing atrial fibrillation and significantly enlarged left atrial diameter (45 mm/m² indexed) rendered her ineligible for ablation. Therefore, we elected to perform left atrial appendage closure alone, without concomitant ablation, in order to minimize operative time and reduce procedural invasiveness, given the patient’s overall frailty and comorbidities.

**Figure 2:**
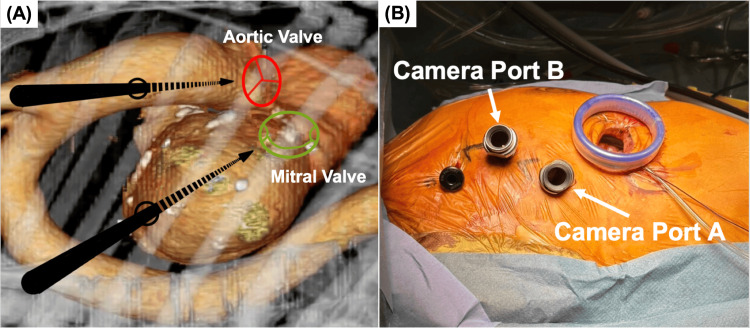
Dual-Camera Port Setup and Intraoperative Port Configuration (A) Preoperative contrast-enhanced computed tomography demonstrating assessment of optimal access trajectories for en face endoscopic visualization of both the aortic and mitral valves. Arrows indicate the estimated camera port entry angles to achieve this view. (B) Intraoperative view of the port configuration, showing a 4-cm main working incision, two camera ports, and an instrument port.

Surgery was indicated for the treatment of heart failure and prevention of recurrent embolic events. Given her small body surface area (BSA 1.2 m²) and low activities of daily living, median sternotomy was considered overly invasive, and a totally endoscopic approach was chosen. Preoperative CT revealed that the direct visualization angles for the aortic and mitral valves differed significantly and that a single camera port would not allow for optimal maneuverability (Figure 2A). Therefore, two separate camera ports were placed to allow individualized approaches for each valve.　

Under general anesthesia using a double-lumen endotracheal tube, the patient's right arm was elevated, and a 4-cm skin incision was made in the fourth intercostal space. A 10-mm Camera Port A was placed at the midclavicular line in the fourth intercostal space (Camera Port A), and the other was positioned at the anterior axillary line in the third intercostal space (Camera Port B). A 5-mm working port was inserted into the second intercostal space (Figure 2B).

**Figure 3:**
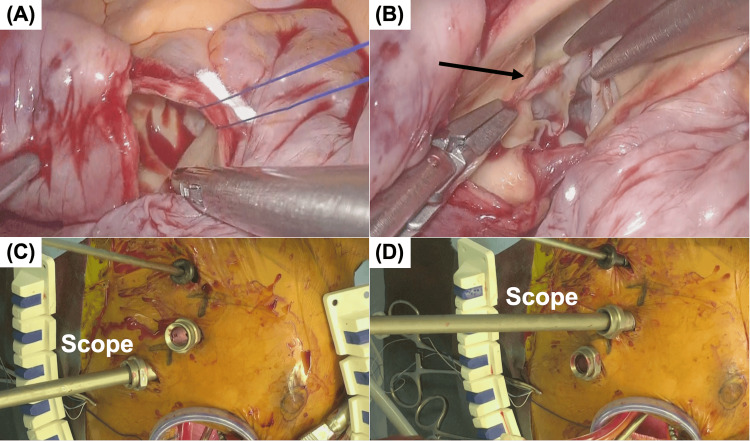
Endoscopic Views Through Different Camera Ports (A) Endoscopic view of the aortic valve through Camera Port A. Only a partial portion of the valve cusps is visible. (B) Endoscopic view of the aortic valve through Camera Port B, demonstrating adequate exposure of the entire valve. (C) Intraoperative photograph showing insertion of the endoscope through Camera Port A. (D) Intraoperative photograph showing insertion of the endoscope through Camera Port B. The arrow indicates the papillary fibroelastoma

Cardiopulmonary bypass (CPB) was established via femoral arterial and venous cannulation. A 3D endoscope (Karl Storz, Tuttlingen, Germany) was introduced through Camera Port A. The ascending aorta was clamped, and cardioplegia was administered to achieve cardiac arrest. The mitral valve was approached through a right-sided left atriotomy. A thrombus was found in the left atrial appendage and was excised. The mitral valve showed commissural fusion and leaflet calcification. The calcified portions of the anterior and posterior leaflets were resected, and a mitral bioprosthetic valve (Edwards Lifesciences, Irvine, CA, USA) was implanted using a non-everting mattress suture technique. The left atrium was closed in a single layer, and the left atrial appendage was occluded using an AtriClip (AtriCure Inc., Westchester, OH, USA). Subsequently, the aorta was transversely incised to examine the aortic valve. Owing to the limited visualization, the camera was switched to Camera Port B, resulting in improved exposure. A papillary fibroelastoma was identified on the right coronary cusp and successfully excised (Figure 3).

The aortic incision was closed in two layers. The patient was weaned from CPB uneventfully. The total CPB time was 133 minutes, and the aortic cross-clamp time was 98 minutes (Video 1).

**Video 1:** Totally Endoscopic Double-Valve Surgery Using a Dual-Camera Port Setup Totally endoscopic mitral valve replacement, left atrial appendage closure, thrombus removal from the left atrial appendage, and papillary fibroelastoma excision performed using a dual-camera port setup.

The postoperative course was uneventful. Transthoracic echocardiography confirmed good prosthetic valve function and normal aortic valve morphology. The patient was discharged on postoperative day seven. At six months postoperatively, the patient maintained NYHA functional class I status with no recurrence of heart failure symptoms. Follow-up transthoracic echocardiography demonstrated stable and favorable prosthetic valve function. No thromboembolic or bleeding complications, including stroke, were observed during the follow-up period. The BNP level decreased to 230 pg/mL. Overall, the postoperative course was uneventful and favorable.

## Discussion

Endoscopic cardiac surgery has demonstrated favorable early postoperative outcomes owing to its minimally invasive approach [1-3]. There have been remarkable advances in endoscopic surgical instruments, with reports of shortened cardiopulmonary bypass times through the use of 3D endoscopes as well as high success rates and improved safety thanks to the advent of left atrial appendage closure devices [4,5]. In recent years, their application in mitral valve repair has gained widespread acceptance, yielding promising results [2]. However, total endoscopic aortic valve replacement (AVR) and combined procedures involving both the aortic and mitral valves remain limited in the selection of centers [3]. A major technical challenge in such procedures is the different optimal visualization angles for each valve [1,2]. Although the aortic and mitral valves are anatomically adjacent, the angles required for a direct endoscopic view differ significantly, rendering the single-port approach suboptimal. Typically, port placement for AVR is one intercostal space higher than that for mitral valve surgery [1,2]. In our case, we addressed this issue by performing a simultaneous total endoscopic procedure using two strategically placed camera ports, allowing for independent approaches to each valve. Preoperative contrast-enhanced computed tomography has been reported to facilitate the determination of the optimal fluoroscopic angulation for valve visualization during catheter-based interventions for structural heart disease [8]. Preoperative computed tomography confirmed that optimal visualization of both valves could not be achieved using a single camera port.

We have previously reported a technique for enhancing the operability of endoscopic internal thoracic artery harvesting during minimally invasive coronary artery bypass surgery by adjusting the camera port position according to the anatomical location of the target segment [9]. By implementing two separate camera ports, the exposure and maneuverability of both valve procedures were significantly improved. 

Although papillary fibroelastomas do not have universally accepted surgical indications, previous reports have noted their association with embolic stroke [10]. Considering the patient's prior cerebrovascular events, surgical intervention was deemed appropriate.

Importantly, the patient was frail and had a small body habitus, rendering median sternotomy particularly invasive. Previous studies have reported poor outcomes after sternotomy in frail patients, highlighting the need for minimally invasive strategies [7]. Our approach, which involves the addition of only one extra port without robotic assistance, allows for a low-cost and simplified technique. The patient possessed a history of persistent atrial fibrillation for over 10 years, with a markedly enlarged left atrial index of 45 mm/m². Given the chronicity of atrial fibrillation, the extent of atrial remodeling, and concerns regarding prolonged operative time, a concomitant Maze procedure was not performed in order to shorten the operative time as much as possible.

The operative time remained within acceptable limits, and the patient recovered without any complications. This case demonstrates that, with meticulous preoperative planning and intraoperative adaptability, totally endoscopic simultaneous aortic and mitral valve surgeries can be performed safely and effectively.

## Conclusions

The advancement of surgical technologies, including high-definition 3D endoscopes and left atrial appendage occlusion devices, has significantly enhanced the safety, precision, and feasibility of totally endoscopic cardiac procedures. In particular, the dual-camera port strategy presented here resolves the technical challenge of conflicting visualization angles between the aortic and mitral valves, thereby improving exposure, instrument handling, and overall procedural control.

This minimally invasive approach is especially advantageous for patients where catheter-based therapies are contraindicated and surgical intervention remains necessary. In the present case, given the patient’s frailty, anemia, and advanced age, a simplified surgical strategy was adopted. AtriClip placement was chosen over a full Maze procedure to minimize operative burden while still addressing the risk of thromboembolism.

This case highlights the importance of tailoring surgical strategies to individual patient profiles and underscores the role of integrated technological solutions in advancing endoscopic cardiac surgery. Broader adoption of such techniques may be facilitated through the accumulation of further cases and continued efforts toward procedural standardization.
